# Supplementary material for: Molecular Docking and Site-Directed Mutagenesis of GH49 Family Dextranase for the Preparation of High-Degree Polymerization Isomaltooligosaccharide
Source: Biomolecules. 2023 Feb 6;13(2):300. doi: 10.3390/biom13020300 (PMC9953027; doi:10.3390/biom13020300)
Supplement: Supplementary file 1 [file biomolecules-13-00300-s001.zip › biomolecules-2158057-SI.pdf]

Molecular Docking and Site-Directed Mutagenesis of GH49 Family Dextranase for the Preparation of High-Degree Polymerization Isomaltooligosaccharide

Huanyu Wang [1,2], Qianru Lin [1,2], Mingwang Liu [1,2], Wen Ding [1,2], Nanhai Weng [1,2], Hao Ni [1,2], Jing Lu [1,2,*], Mingsheng Lyu [1,2] and Shujun Wang [1,2,*]

1 Jiangsu Key Laboratory of Marine Bioresources and Environment/Jiangsu Key Laboratory of Marine Biotechnology, Jiangsu Ocean University, Lianyungang 222005, China
2 Co-Innovation Center of Jiangsu Marine Bio-Industry Technology, Jiangsu Ocean University, Lianyungang 222005, China
* Correspondence: jinglu@jou.edu.cn (J.L.); sjwang@jou.edu.cn (S.W.)

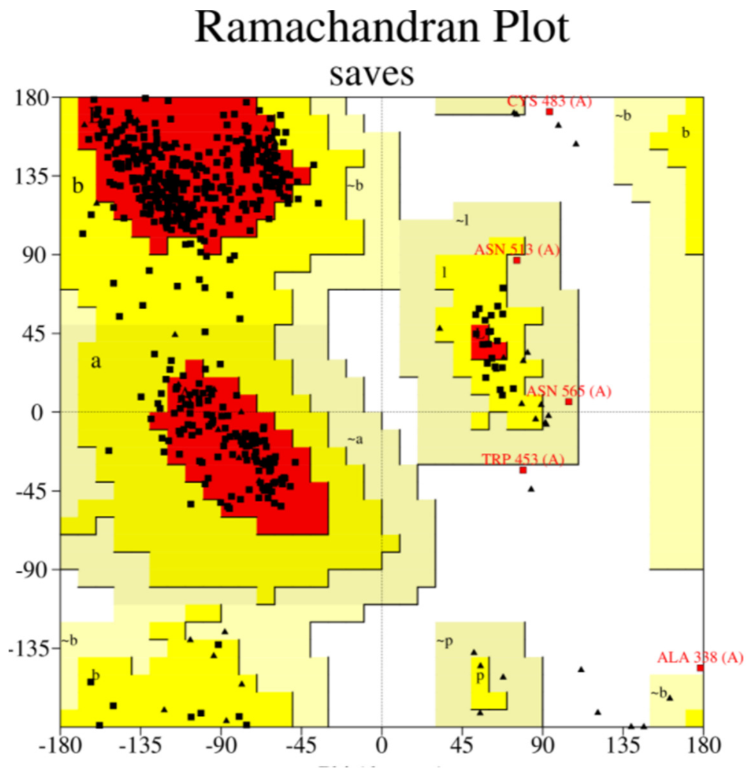

Figure S1. PsDex1711 model assessment of the Ramachandran.

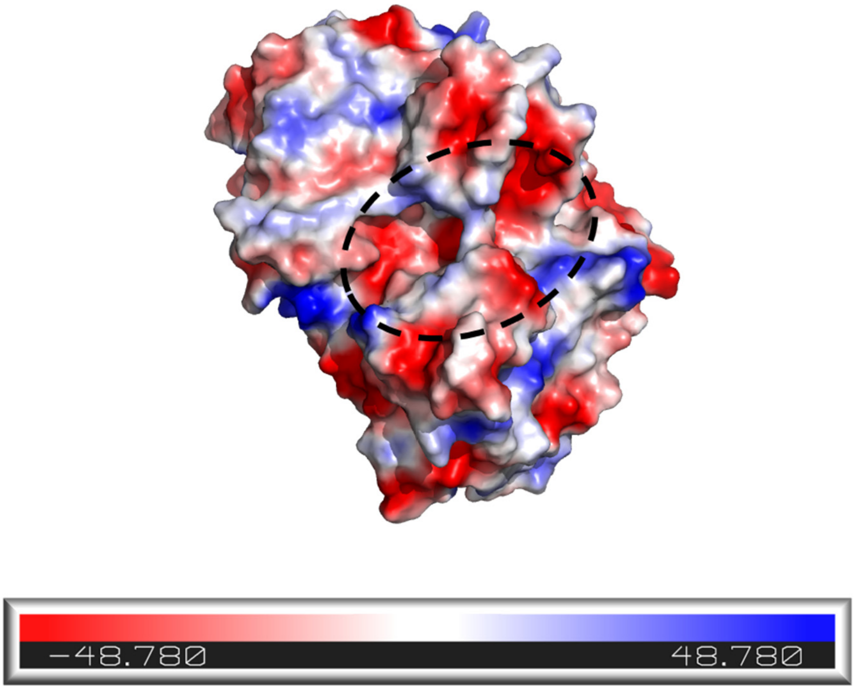

Figure S2. PsDex1711 electrostatic potential map. The black dashed line is the catalytic cleft.

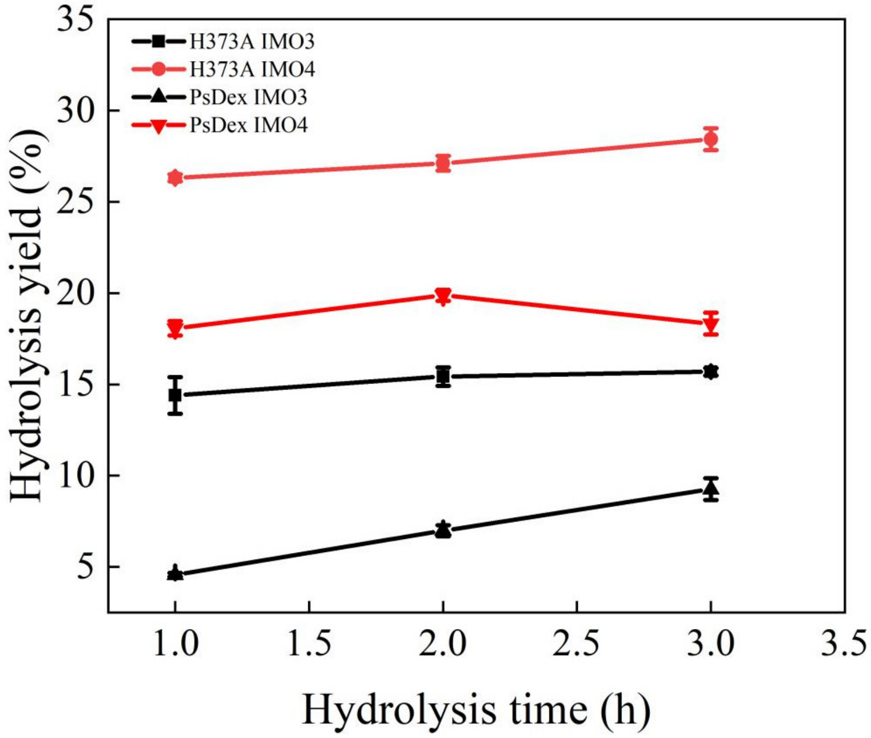

Figure S3. Analysis of the yield of H373A hydrolysate.

Table S1. Primer design of the mutant enzyme.

	Primer （5′—3′）
K427A-F	CAATGACGACGTCCTGGCGCTCTACCACAGCAACG
K427A-R	CGTTGCTGTGGTAGAGCGCCAGGACGTCGTCATTG
H373A-F	CAGCGAGCCGCCCTACGCCTCCTTCGTCATCTATG
H373A-R	CATAGATGACGAAGGAGGCGTAGGGCGGCTCGCTG
N445A-F	GATCTGGAAGAACGAGGCCGGCCCGGTTATCCAAT
N445A-R	ATTGGATAACCGGGCCGGCCTCGTTCTTCCAGATC
S431A-F	TCCTGAAGCTTACCACGCCAACGTGACGGTGGATA
S431A-R	TATCCACCGTCACGTTGGCGTGGTAAGCTTCAGGA
Q402A-F	CGGCAGTTGG TACTGGGCGACCGACGGCATGGAAC
Q402A-R	GTTCCATGCCGT CGGTCGCCCAGTACCAACTGCCG

Table S2. Primer design of the saturated mutant enzyme.

	Primer （5′—3′）
H373R-F	GCGAGCCGCCCTACCGCTCCTTCGTC
H373R-R	CGGTAGGGCGGCTCGCTGATGGTGAT
H373F-F	CAGCGAGCCGCCCTACTTCTCCTTCGTCATCTATG
H373F-R	CATAGATGACGAAGGAGAAGTAGGGCGGCTCGCTG
H373C-F	CAGCGAGCCGCCCTACTGCTCCTTCGTCATCTATG
H373C-R	CATAGATGACGAAGGAGCAGTAGGGCGGCTCGCTG
H373G-F	CAGCGAGCCGCCCTACGGCTCCTTCGTCATCTATG
H373G-R	CATAGATGACGAAGGAGCCGTAGGGCGGCTCGCTG
H373Q-F	CGAGCCGCCCTACCAGTCCTTCGTCA
H373Q-R	CTGGTAGGGCGGCTCGCTGATGGTGA
H373D-F	AGCGAGCCGCCCTACGACTCCTTCGT
H373D-R	CGTAGGGCGGCTCGCTGATGGTGATT
H373E-F	CAGCGAGCCGCCCTACGAGTCCTTCGTCATCTATG
H373E-R	CATAGATGACGAAGGACTCGTAGGGCGGCTCGCTG
H373K-F	CAGCGAGCCGCCCTACAAGTCCTTCGTCATCTATG
H373K-R	CATAGATGACGAAGGACTTGTAGGGCGGCTCGCTG
H373L-F	GCGAGCCGCCCTACCTCTCCTTCGTC
H373L-R	AGGTAGGGCGGCTCGCTGATGGTGAT
H373M-F	CAGCGAGCCGCCCTACATGTCCTTCGTCATCTATG
H373M-R	CATAGATGACGAAGGACATGTAGGGCGGCTCGCTG

H373N-F	AGCGAGCCGCCCTACAACTCCTTCGT
H373N-R	TGTAGGGCGGCTCGCTGATGGTGATT
H373S-F	CAGCGAGCCGCCCTACAGCTCCTTCGTCATCTATG
H373S-R	CATAGATGACGAAGGAGCTGTAGGGCGGCTCGCTG
H373Y-F	AGCGAGCCGCCCTACTACTCCTTCGT
H373Y-R	AGTAGGGCGGCTCGCTGATGGTGATT
H373T-F	CAGCGAGCCGCCCTACACCTCCTTCGTCATCTATG
H373T-R	CATAGATGACGAAGGAGGTGTAGGGCGGCTCGCTG
H373I-F	CAGCGAGCCGCCCTACATCTCCTTCGTCATCTATG
H373I-R	CATAGATGACGAAGGAGATGTAGGGCGGCTCGCTG
H373W-F	CAGCGAGCCGCCCTACTGGTCCTTCGTCATCTATG
H373W-R	CATAGATGACGAAGGAACCGTAGGGCGGCTCGCTG
H373P-F	GCGAGCCGCCCTACCCCTCCTTCGTC
H373P-R	GGGTAGGGCGGCTCGCTGATGGTGAT
H373V-F	CAGCGAGCCGCCCTACGTCTCCTTCGTCATCTATG
H373V-R	CATAGATGACGAAGGAGACGTAGGGCGGCTCGCTG

Table S3. Primer design for the validation of site-specific mutases.

	Primer （5′—3′）
P371R-F	CATCAGCGAGCCGCCCCGCCACTCCTTCGTCATCT
P371R-R	AGATGACGAAGGAGTGGCGGGGCGGCTCGCTGATG
Y372R-F	CCATCAGCGAGCCGCGCCGCCACTCC
Y372R-R	CGCGGCTCGCTGATGGTGATTCCCTC
S374R-F	CGAGCCGCCCTACCACCGCTTCGTCATCTATGGGC
S374R-R	GCCCATAGATGACGAAGCGGTGGTAGGGCGGCTCG
F375R-F	GCCGCCCTACCACTCCCGCGTCATCTATGGGCCCCA
F375R-R	TGGGGCCCATAGATGACGCGGGAGTGGTAGGGCGGC

Table S4. Primer design for the two-site mutas.

	Primer （5′——3′）
H373R/N445A-F	ATCCACGCCCGCATGTATTGGAAGGACGTCAAA
H373R/N445A-R	ATACATGCGGGCGTGGATGACGTCAGTGTTGGTTAC
H373R/N445R-F	ATCCACCGCCGCATGTATTGGAAGGACGTCAAA
H373R/N445R-R	ATACATGCGGCGGTGGATGACGTCAGTGTTGGTTAC
H373R/N445F-F	TCATCCACTTCCGCATGTATTGGAAGGACGTCA
H373R/N445F-R	ACATGCGGAAGTGGATGACGTCAGTGTTGGTTAC
H373R/N445C-F	ATCCACTGCCGCATGTATTGGAAGGACGTCAAA
H373R/N445C-R	ATACATGCGGCAGTGGATGACGTCAGTGTTGGTTAC
H373R/N445G-F	ATCCACGGCCGCATGTATTGGAAGGACGTCAAA
H373R/N445G-R	ATACATGCGGCCGTGGATGACGTCAGTGTTGGTTAC

H373R/N445Q-F	ATCCACCAGCGCATGTATTGGAAGGACGTCAA
H373R/N445Q-R	TACATGCGCTGGTGGATGACGTCAGTGTTGGTTAC
H373R/N445D-F	ATCCACGACCGCATGTATTGGAAGGACGTCAA
H373R/N445D-R	TACATGCGGTCGTGGATGACGTCAGTGTTGGTTAC
H373R/N445E-F	ATCCACGAGCGCATGTATTGGAAGGACGTCAA
H373R/N445E-R	TACATGCGCTCGTGGATGACGTCAGTGTTGGTTAC
H373R/N445K-F	ATCCACAAGCGCATGTATTGGAAGGACGTCAA
H373R/N445K-R	TACATGCGCTTGTGGATGACGTCAGTGTTGGT
H373R/N445L-F	ATCCACCTCCGCATGTATTGGAAGGACGTCAAA
H373R/N445L-R	ATACATGCGGAGGTGGATGACGTCAGTGTTGGTTAC
H373R/N445M-F	CATCCACATGCGCATGTATTGGAAGGACGTCAA

H373R/N445M-R	TACATGCGCATGTGGATGACGTCAGTGTTGGTT
H373R/N445S-F	ATCCACAGCCGCATGTATTGGAAGGACGTCAA
H373R/N445S-R	TACATGCGGCTGTGGATGACGTCAGTGTTGGT
H373R/N445Y-F	TCATCCACTACCGCATGTATTGGAAGGACGTC
H373R/N445Y-R	CATGCGGTAGTGGATGACGTCAGTGTTGGTTAC
H373R/N445T-F	ATCCACACCCGCATGTATTGGAAGGACGTCAA
H373R/N445T-R	TACATGCGGGTGTGGATGACGTCAGTGTTGGT
H373R/N445I-F	CATCCACATCCGCATGTATTGGAAGGACGTCA
H373R/N445I-R	ACATGCGGATGTGGATGACGTCAGTGTTGGTT
H373R/N445W-F	ATCCACTGGCGCATGTATTGGAAGGACGTCAA
H373R/N445W-R	TACATGCGCCAGTGGATGACGTCAGTGTTGGTTAC

H373R/N445P-F	ATCCACCCCCGCATGTATTGGAAGGACGTCAAA
H373R/N445P-R	ATACATGCGGGGGTGGATGACGTCAGTGTTGGTTAC
H373R/N445V-F	ATCCACGTCCGCATGTATTGGAAGGACGTCAAA
H373R/N445V-R	ATACATGCGGACGTGGATGACGTCAGTGTTGGTTAC
H373R/N445H-F	ATCCACCACCGCATGTATTGGAAGGACGTCAA
H373R/N445H-R	TACATGCGGTGGTGGATGACGTCAGTGTTGGTTAC
